# Vpr14-88-Apobec3G Fusion Protein Is Efficiently Incorporated into Vif-Positive HIV-1 Particles and Inhibits Viral Infection

**DOI:** 10.1371/journal.pone.0001995

**Published:** 2008-04-16

**Authors:** Zhujun Ao, Zhe Yu, Lina Wang, Yingfeng Zheng, Xiaojian Yao

**Affiliations:** Laboratory of Molecular Human Retrovirology, Department of Medical Microbiology, Faculty of Medicine, University of Manitoba, Winnipeg, Canada; AIDS Research Center, Chinese Academy of Medical Sciences and Peking Union Medical College, China

## Abstract

**Background:**

APOBEC3G (A3G), a deoxycytidine deaminase, is a potent host antiviral factor that can restrict HIV-1 infection. During Vif-negative HIV-1 replication, A3G is incorporated into HIV-1 particles, induces mutations in reverse transcribed viral DNA and inhibits reverse transcription. However, HIV-1 Vif counteracts A3G's activities by inducing its degradation and by blocking its incorporation into HIV-1 particles. Thus, it is interesting to elucidate a mechanism that would allow A3G to escape the effects of Vif in order to rescue its potent antiviral activity and to provide a possible novel therapeutic strategy for treating HIV-1 infection.

**Methods and Findings:**

In this study, we generated an R88-A3G fusion protein by fusing A3G to a virion-targeting polypeptide (R14-88) derived from HIV-1 Vpr protein and compared its antiviral effects relative to those of HA-tagged native A3G (HA-A3G). Our study showed that transient expression of the R88-A3G fusion protein in both Vif^−^ and Vif^+^ HIV-1 producing cells drastically inhibited viral infection in HeLa-CD4-CCR5-cells, CD4^+^ C8166 T cells and human primary PBMCs. Moreover, we established CD4^+^ C8166 T cell lines that stably express either R88-A3G or HA-A3G by transduction with VSV-G-pseudotyped lentiviral vector that harbor expression cassettes for R88-A3G or HA-A3G, respectively, and tested their susceptibility to Vif^+^ HIV-1 infection. Our results clearly reveal that expression of R88-A3G in transduced CD4^+^ C8166 cells significantly blocked Vif^+^ HIV-1 infection. In an attempt to understand the mechanism underlying the antiviral activity of R88-A3G, we demonstrated that R88-A3G was efficiently incorporated into viral particles in the presence of Vif. Moreover, PCR analysis revealed that R88-A3G significantly inhibited viral cDNA synthesis during the early stage of Vif^+^ virus infection.

**Conclusions:**

Our results clearly indicate that R88 delivers A3G into Vif^+^ HIV-1 particles and inhibits infectivity and spread of the virions among CD4^+^ T cells. This study provides evidence for an effective strategy to modify a host protein with innate anti-HIV-1 activity and rescue its potent anti-HIV potential in the presence of Vif. Further characterization and optimization of this system may lead to the development of an effective therapeutic approach against HIV-1 infection.

## Introduction

Human immunodeficiency virus type 1 (HIV-1) infection of primary CD4^+^ T cells, macrophages and some immortalized T cell lines requires the HIV-1 encoded viral infectivity factor (Vif) protein. In the absence of Vif protein, apolipoprotein B mRNA editing enzyme, catalytic polypeptide-like 3G (APOBEC3G; hereafter referred to as A3G), which is a cellular cytidine deaminase, was found to interfere with the replication of retroviruses, including HIV-1 [1]. A3G is efficiently incorporated into viral particles, associates with the HIV-1 reverse transcription complex (RTC), and interrupts HIV infectivity by introducing dC-to-dU mutations in the minus viral DNA strand during reverse transcription [2]–[6]. In addition to its deaminase activity, A3G directly inhibits viral reverse transcription [7], [8]. These previous observations highlight the multifaceted anti-HIV activities of A3G during HIV-1 replication.

In activated T lymphocytes, A3G is packaged into the progeny virus through interactions with the NC domain of Gag and/or with the viral RNA during virion assembly [9]–[16]. However, during wild-type HIV-1 infection, the antiviral effects of A3G are blocked by Vif, which decreases incorporation of A3G into virions by reducing the intracellular level of A3G through accelerating ubiquitination and proteasomal degradation of A3G [6], [17]–[23]. In addition, previous studies suggest that Vif may act as an effective barrier to completely block targeting of A3G into virions, based on the observation that, even though a low level of A3G was detected in Vif^+^ HIV producing cells, the progeny virions remained infectious [6], [22], [24]–[26]. Thus, breaking through Vif's barrier and successfully targeting A3G into virions may promote inactivation of HIV-1 and eliminate its infectivity.

Given that A3G exerts potent anti-HIV activity which is neutralized by the HIV-1 Vif protein, characterization of the A3G-Vif interaction is of considerable interest, as it provides a target for novel therapeutic strategies against HIV-1 infection. Recent studies have shown that a single amino-acid substitution of an aspartic acid residue to a lysine at position of 128 of A3G abrogated its interaction with HIV-1 Vif and rescued A3G's antiviral activity [27]–[30]. Furthermore, Huthoff *et al.,* employed a molecular genetic approach to map a 3 amino-acid motif, comprised of aspartic acid-proline-aspartic acid (DPD), at amino-acid positions 128 to 130 of A3G that is a crucial region for the interaction between A3G and HIV-1 Vif [31]. In addition, a 4 amino-acid region (YYFW) adjacent to the N-terminus of the DPD motif of A3G has been identified as an important determinant for virion packaging of A3G. Such an intimate alignment of these two functional domains within A3G raises the possibility that disruption of the A3G/Vif interaction by targeting of the DPD motif with pharmaceutical agents may simultaneously interfere with recognition of the A3G packaging signal, and affect its antiviral activity. Therefore, developing novel strategies to efficiently target A3G into virions by allowing it to escape from Vif's blockage will broaden our current arsenal of anti-HIV therapies.

HIV-1 Vpr, a viral auxiliary protein, is efficiently incorporated *in trans* into HIV particles, through its interaction with the p6 domain of the Gag precursor polyprotein [32]–[36]. Its high incorporation efficiency into HIV-1 virions has enabled Vpr to deliver heterologous molecules, as Vpr fusion proteins, into the virus, thereby interfering with viral infectivity or complementing a defective virus [37]–[44]. It has also been shown that a virion-incorporation peptide R14-88 derived from Vpr can efficiently direct heterologous enzymatic proteins, such as chloramphenicol acetyltransferase, into HIV-1 particles in a p6-dependent manner [41]. In this study, we generated an R14-88-pcs-A3G (R88-A3G) fusion protein by fusing A3G to R14-88 and investigated its anti-HIV effect in the presence of Vif. Our study has shown that co-expression of R88-A3G with Vif^+^ HIV-1 provirus (HxBru-Vif^+^) drastically inhibited the infectivity of progeny virus in HeLa-CD4-CCR5-β-Gal cells, CD4+ C8166 cells and human primary PBMCs. The same inhibitory effect was also observed in susceptible CD4^+^ C8166 T cell lines that stably express R88-A3G. Moreover, we found that R88-A3G was efficiently incorporated into viral particles and significantly inhibited reverse transcription during the early stage of Vif^+^ virus replication. These results support the hypothesis that the R88-A3G fusion protein acts as a potent antiviral agent against HIV-1 infection.

## Results

### Expression of R88-A3G efficiently inhibits HIV-1 infection regardless of the presence of Vif

**Figure 1:**
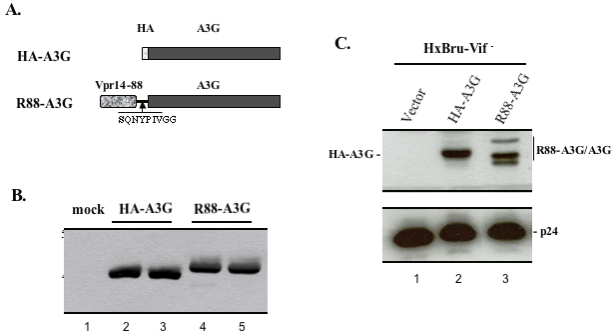
R88-A3G was expressed in viral producing cells and cleaved by viral protease. A). Schematic representation of constructs of HA-A3G and R88-A3G. HA-tag or Vpr14-88 was fused in frame to the N-terminus of Apobec3G (A3G). An HIV-1 protease cleavage site (PCS) was inserted between R88 and A3G. B). HA-A3G or R88-A3G was expressed in 293T cells. 293T cells were transfected with the HA-A3G or R88-A3G expressor. After 48 hours, cell lysates were immunoprecipitated with a mixture of anti-HA and anti-Vpr antibodies followed by Western blotting with anti-A3G antibody. C). Cleavage of R88-A3G in virus produced from HIV-1 expressing cells. 293T cells were cotransfected with HxBru-Vif^−^ provirus and the HA-A3G or R-A3G expressing plasmid. After 48 hours, produced viruses were pelleted from supernatant by ultracentrifugation through a 20% sucrose cushion, lysed, and directly loaded onto 10% SDS-PAGE followed by Western blotting with anti-A3G polyclonal antibody (upper panel) or with anti-p24 monoclonal antibody (lower panel). The positions of R88-A3G and the cleaved product of A3G are indicated at right side of gel.

We initially sought to develop a strategy to antagonize the Vif-mediated blockade of A3G incorporation, and subsequently to inhibit HIV infection. A fusion protein termed R88-A3G was generated by fusing full-length A3G to the C-terminus of a virion-incorporation domain (R14-88) of HIV-1 Vpr [41] (Fig. 1A). An HIV-1 protease cleavage site (SQNY/PIV) was inserted between R14-88 and A3G in order to release the native A3G protein in the virion through a specific cleavage mediated by HIV-1 protease. In parallel, an HA-A3G-expressorwas constructed as a control. The expression of HA-A3G and R88-A3G fusion proteins was monitored by Western blotting with the anti-A3G antibody in 293T cells transfected with either R88-A3G or HA-A3G expressor alone, or co-transfected with an HIV-1 HxBru-Vif^−^ provirus. Our results show that, in the absence of HIV-1 expression, the R88-A3G and HA-A3G proteins were detected in transfected 293T cells with approximate molecular weights of 47 kDa and 43 kDa, respectively (Fig. 1B). In the HIV-1 expressing cells, while HA-A3G retained the same molecular weight, the majority of the R88-A3G fusion proteins had molecular weights ranging between approximately 39–40 kDa (Fig. 1C), indicating that the R88-A3G fusion protein was efficiently cleaved by HIV-1 protease.

**Figure 2:**
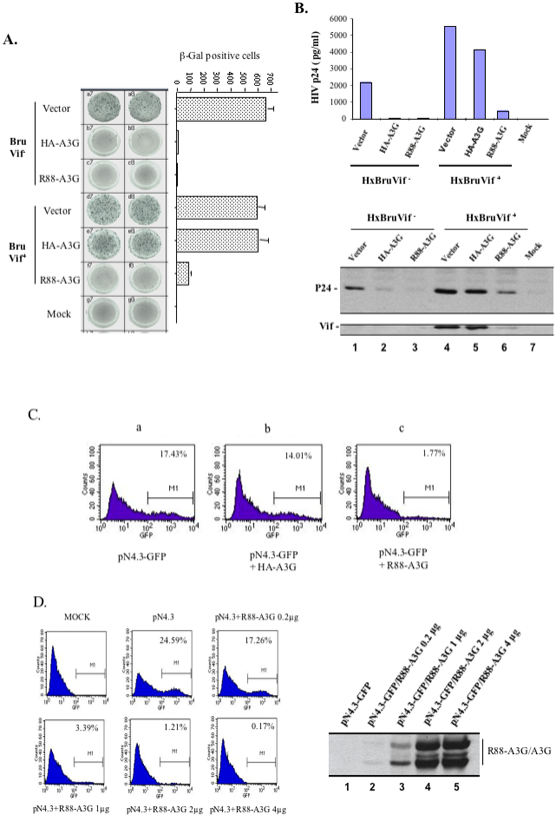
Expression of R88-A3G in HIV-1 producing cells inhibits virus infectivity in the presence of Vif. Viruses were produced from 293T cells transfected with 3 µg of HIV-1 HxBru-Vif^+^ or HxBru-Vif^−^ with 4 µg of HA-A3G or R-A3G expressor. Then, equal amounts of produced viruses (as adjusted by the Gag-p24 level) were used to infect HeLa-CD4-CCR5-β-Gal cells and CD4+ C8166 T cells. A). At 48 hours post-infection, HIV-1 infected cells were detected by MAGI assay (left panel) and counted by Elispot Reader (right panel). B). At 48 hours post-infection, virus production in of C8166 cells were monitored by measurement of HIV-1 Gag-p24^gag^ antigen in the supernatants with p24 ELISA assay (upper panel). Cell-associated HIV-1 Gag-p24 (middle panel) and Vif (lower panel) were detected by Western blotting with anti-p24 or anti-Vif antibodies, respectively. C). pNL4.3-GFP viruses were first produced from 293T cells co-transfected by pNL4.3/GFP provirus with HA-A3G or R88-A3G. Then, equal amounts of pNL4.3-GFP viruses were used to infect C8166 T cells. At 72 hours post-infection, the percentage of infected (GFP-positive) cells was measured by FACS analysis. D). Dose-dependent effect of R88-A3G on HIV-1 infectivity. Different amounts of R88-A3G plasmid (0, 0.2, 1, 2, 4 µg) and pNL4.3-GFP proviral DNA (3 µg) were used to co-transfect 293T cells. Viruses from each transfected cell culture were collected and equal Gag-p24 amounts of viruses were used to infect C8166 T cells. 72 hours later, percentage of infected (GFP-positive) cells was measured by FACS analysis (left panel). Meanwhile, the expression of R88-A3G in the corresponding transfected 293T cells was detected by Western blotting with anti-A3G antibody (right panel).

To determine whether R88-A3G exhibits anti-HIV activity in the presence of Vif, we co-transfected 293T cells with R88-A3G or HA-A3G plasmid and either Vif^−^ or Vif^+^ HIV-1 provirus (HxBru-Vif^−^ or HxBru-Vif^+^). At 48 hours post-transfection, the progeny viruses were collected and equal amounts of the viruses (adjusted by HIV-1 Gag-p24 levels) were used to infect HeLa-CD4-CCR5-β-Gal cells (Fig. 2A) and CD4+ C8166 T cells (Fig. 2B). At 2 days post-infection, the HIV-1 infection level was monitored by a MAGI assay [45] in the infected cells or by the quantification of HIV-1 Gag-p24 antigen in the supernatants. As expected, expression of HA-A3G disrupted the infectivity of Vif^−^ viruses, but had no inhibitory effect on Vif^+^ virus infection (Fig. 2A and B). Interestingly, the infectivity of the virus produced from R88-A3G expressing 293T cells was drastically inhibited regardless of Vif expression (Fig. 2A and B), indicating that R88-A3G exhibited potent anti-HIV activity by overwhelming Vif-mediated effects.

To further investigate whether R88-A3G exerts an antiviral effect on other HIV-1 strains, we produced pNL4.3-GFP viruses by co-transfecting pNL4.3/GFP+/Vif+ provirus with either HA-A3G or R88-A3G expressor in 293T cells. The level of infection by progeny viruses was analyzed in CD4+ C88166 T cells by determination of the percentage of infected (GFP-positive) cells with FACS analysis. Our results clearly showed that expression of the R88-A3G fusion protein, but not HA-A3G, efficiently blocked Vif^+^ pNL4.3 virus infection (Fig. 2C). Furthermore, analysis of cells co-transfected with the R14-88 peptide and provirus indicated that R14-88 did not have an inhibitory effect by itself (data not shown). Moreover, a dose-dependent effect of R88-A3G was evaluated. Our results clearly showed that the antiviral effect of R88-A3G against pNL4.3 virus infection was specific to A3G and was dose-dependent, since enhancement of the inhibitory effect of R88-A3G on virus infection (Fig. 2D, left panel) was achieved by increased R88-A3G expression in virus producing cells (Fig. 2D, right panel).

### Analysis of the stability and virus-incorporation of R88-A3G in the presence of Vif

**Figure 3:**
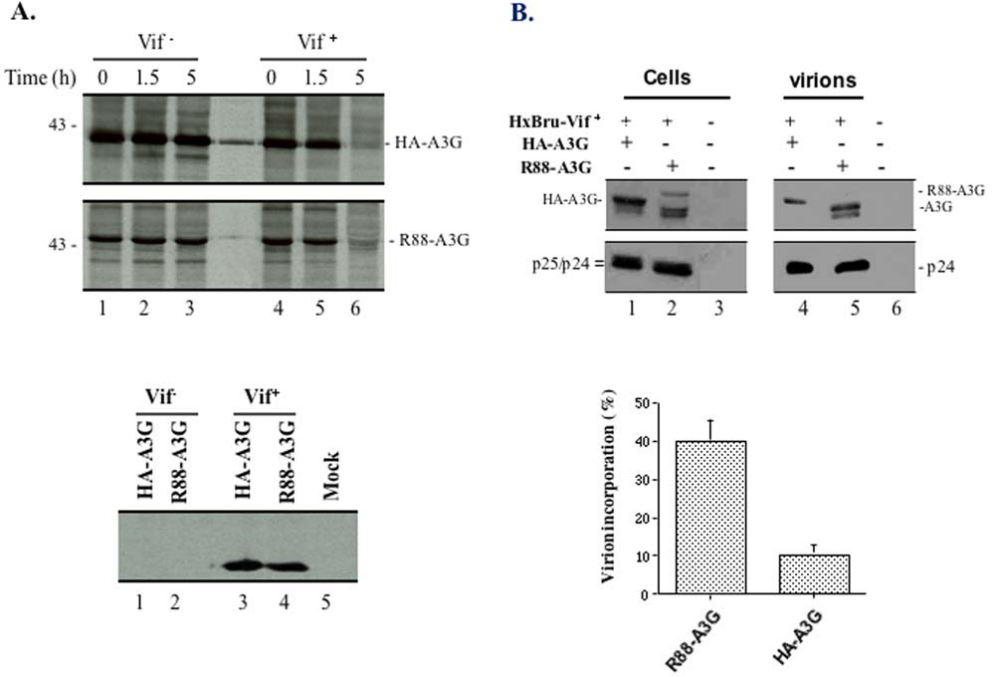
R88-A3G is sensitive to Vif-induced degradation, but is efficiently incorporated into Vif^+^ HIV-1 particles. A). Effect of Vif on the degradation of R88-A3G. HA-A3G or R88-A3G expressor was transfected alone or co-transfected with pcDNA-hVif expressor in 293T cells. At 48 hour post-transfection, cells were pulse radiolabeled with [_35_S]-methionine for 30 min, and labeled cells were collected and lysed at 0, 1.5 and 5 hours after pulse-labeling. Then, the level of HA-A3G in each lysed-cell sample was detected by anti-HA immunoprecipitation (upper panel). The level of R88-A3G in each lysed-cell sample was evaluated by anti-Vpr immunoprecipitation (middle panel). To detect HIV-1 Vif expression in different transfected cell samples, an aliquot of transfected cells from each culture was lysed and directly loaded onto 12.5% SDS-PAGE followed by Western blotting with anti-Vif antibody (lower panel). B). R88-A3G was efficiently incorporated into viral particles in the presence of Vif. 293T cells were co-transfected with HxBru-Vif+ (3ug) and HA-A3G or R88-A3G expressor (2ug). After 48 hours cells were collected and the produced virus particles were collected from supernatant by ultracentrifugation through a 20% sucrose cushion. Both cell and virus lysate samples were directly loaded onto a 12% SDS-PAGE gel and analyzed by Western blotting with rabbit anti-A3G and anti-p24, as indicated (upper panel). The ratio of R88-A3G or HA-A3G incorporation into the viral particle relative to the total amount of R88-A3G or HA-A3G was also quantified by laser densitometry (lower panel). The data presented herein are the means and standard deviations from two independent experiments.

To understand the mechanisms whereby R88-A3G, but not HA-A3G, inhibits HIV-1 infection in the presence of Vif, we compared the intracellular expression and virion-incorporation of R88-A3G and HA-A3G. As Vif induces rapid proteasomal degradation of A3G [6], [17]–[23], we wondered whether R88-A3G fusion protein may be more resistant than HA-A3G to Vif-induced degradation. To address this question, the R88-A3G or HA-A3G plasmid was transfected alone or with an HIV-1 Vif plasmid (pcDNA-hVif) [46] into 293T cells. At 48 hours post-transfection, the stability of each protein in 293T cells was analyzed by [_35_S]methionine pulse-chase radiolabeling and immunoprecipitation with anti-HA or anti-Vpr antibodies, respectively. Also, Vif expression was assessed by SDS-PAGE of cell lysates, followed by Western blotting with the anti-Vif antibody. Our results showed that, in the absence of Vif, both HA-A3G and R88-A3G proteins were stable for up to 5 hours (Fig. 3A, lanes 1 to 3). In contrast, expression of Vif (Fig. 3A, lower panel, lanes 3 and 4) induced rapid degradation of both HA-A3G and R88-A3G; more than 95% of the HA-A3G and R88-A3G proteins were degraded by 5 hours (Fig. 3A, upper panel, lanes 4 to 6), suggesting that both R88-A3G and HA-A3G were equally sensitive to Vif-induced degradation.

Based on the above observations, we considered that the remaining non-degraded R88-A3G, but not HA-A3G, could be efficiently incorporated into virus particles in the presence of Vif. Indeed, several previous reports have indicated that, even though a low level of A3G was detected in Vif^+^ HIV producing cells, Vif may act as an effective barrier to prevent A3G from being incorporated virions virus and thereby completely neutralize the antiviral activity of A3G [6], [22], [24]–[26]. To address this hypothesis, we co-transfected 293T cells with the HA-A3G or R88-A3G plasmids and with the HxBru-Vif^+^ provirus. At 48 hours post-transfection, viral particles were collected and concentrated by ultracentrifugation through a 20% sucrose cushion. Cell- and virion-associated HA-A3G, R88-A3G and Gag-p24 were detected by 12% SDS-PAGE of cell and virus lysates followed by Western blotting with the rabbit anti-A3G and anti-p24 antibodies. Data showed that the intracellular level of R88-A3G was obviously lower than that of HA-A3G (Fig. 3B, upper panel, compare lanes 2 and 1, respectively). Since a similarly lowered level of R88-A3G was observed in the absence of Vif, as shown in figure 3A, we conclude that the expression level difference between R88-A3G and HA-A3G is Vif-independent. Intriguingly, the level of R88-A3G was significantly higher than that of HA-A3G in Vif^+^ virions (Fig. 3B, upper panel, compare lanes 5 and 4, respectively). To further compare the incorporation efficiency of R88-A3G and HA-A3G, levels of HA-A3G and R88-A3G in virions were quantified by densitometric scanning analysis of the autoradiogram presented in figure 3B. The levels of virion-associated R88-A3G and HA-A3G were calculated as ratios of virion-associated R88-A3G and HA-A3G protein relative to total protein levels. This ratio was adjusted relative to the level of virion-associated Gag-p24protein, which was employed as an internal control. These data illustrate that the virion-incorporation rate of R88-A3G was four-fold higher than that of HA-A3G (Fig. 3B, lower panel). These results clearly indicate that R88-A3G is efficiently incorporated into virions by evading Vif blockage.

### Expression of R88-A3G does not affect virus maturation, but significantly inhibits Vif^+^ HIV-1 reverse transcription

**Figure 4:**
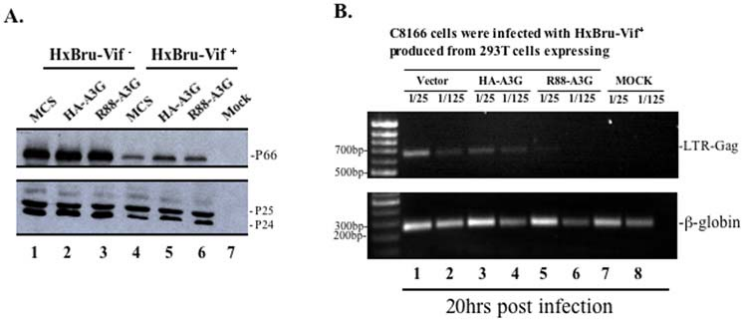
Expression of R88-A3G does not affect virus maturation, but significantly inhibits viral cDNA synthesis. A). R88-A3G does not affect virus maturation. HxBru-Vif^−^ and Vif^+^ viruses produced from 293T cells co-transfected with corresponding provirus and HA-A3G or R88-A3G expressor were pelleted, lysed and processed for immunoprecipitation with human anti-HIV serum. The immunoprecipitates were submitted to 10% SDS-PAGE and analyzed by Western blotting using rabbit anti-anti-RT antibody (upper panel) and anti-p24 antibodies (lower panel). B). R88-A3G inhibits viral cDNA synthesis at early stage of viral infection. Dividing C8166 T cells were infected with equal amounts of different HxBru-Vif^+^ virus stocks, which were produced from 293T cells co-transfected with the provirus and HA-A3G or R88-A3G expressor. At 12 hours post-infection, 1×10^6^ cells were lysed and the total viral DNA was detected by PCR using HIV-1 LTR-Gag primers, as described in Materials and Methods. In parallel, the content of cellular β-globin DNA in each sample were also examined by specific PCR analysis (lower panel).

The above results indicate that R88-A3G is efficiently incorporated into virions despite the presence of Vif. We next addressed how incorporated R88-A3G affects HIV-1 replication. Firstly, we determined whether the presence of R88-A3G affects HIV-1 maturation during virus production. The levels of virion-associated reverse transcriptase and Gag-p24 were visualized by immunoprecipitation of lysed virus-samples with human anti-HIV serum followed by Western blotting with rabbit anti-RT and anti-p24 antibodies. Similar levels of reverse transcriptase and Gag-p24 were detected in Vif^+^ and Vif^−^ viruses regardless of HA-A3G or R88-A3G expression during virus production (Fig. 4A), indicating that presence of HA-A3G or R88-A3G did not affect virus maturation.

Several studies have reported that, in the absence of Vif, encapsidated A3G is capable of inhibiting HIV-1 reverse transcription after virus entry, and that this activity is not related to A3G's deaminase activity [8], [47]–[49]. Since R88-A3G was efficiently incorporated into virions and exerted potent antiviral activity, we postulate that R88-A3G may affect reverse transcription during the early stage of Vif^+^ HIV-1 infection. To test this possibility, we infected C8166 cells with equal amounts HxBru-Vif+ viruses (normalized by virus-associated Gag-p24 levels) generated from 293T cells which were cotransfected with HA-A3G or R88-A3G plasmids. At 12 hours post-infection, total DNA was extracted and processed to detect total viral DNA synthesis by submitted to semi-quantitative PCR analysis using LTR-Gag primers, as described previously [50]. PCR analysis showed that similar levels of viral cDNA were present in C8166 T cells infected with wild type virus or virus produced from HA-A3G-expressing 293T cells, indicating that Vif blocked the anti-HIV activity of HA-A3G (Fig. 4B, compare lanes 3 and 4 to lanes 1 and 2). Strikingly, the reverse transcription level detected for virions containing R88-A3G was drastically reduced (Fig. 4B, lanes 5 and 6). This differential rate of viral cDNA synthesis detected among R88-A3G and wild-type/HA-A3G viruses was not due to intrinsic variation between samples since similar levels of cellular DNA (inferred from amplification of the β-globin gene) were detected for all samples (Fig. 4B, lower panel). Together, this data indicates that virion-associated R88-A3G significantly inhibited reverse transcription during Vif^+^ HIV replication.

### Stable expression of R88-A3G in CD4+ C8166 cells has no effect on cell growth or CD4 expression

**Figure 5:**
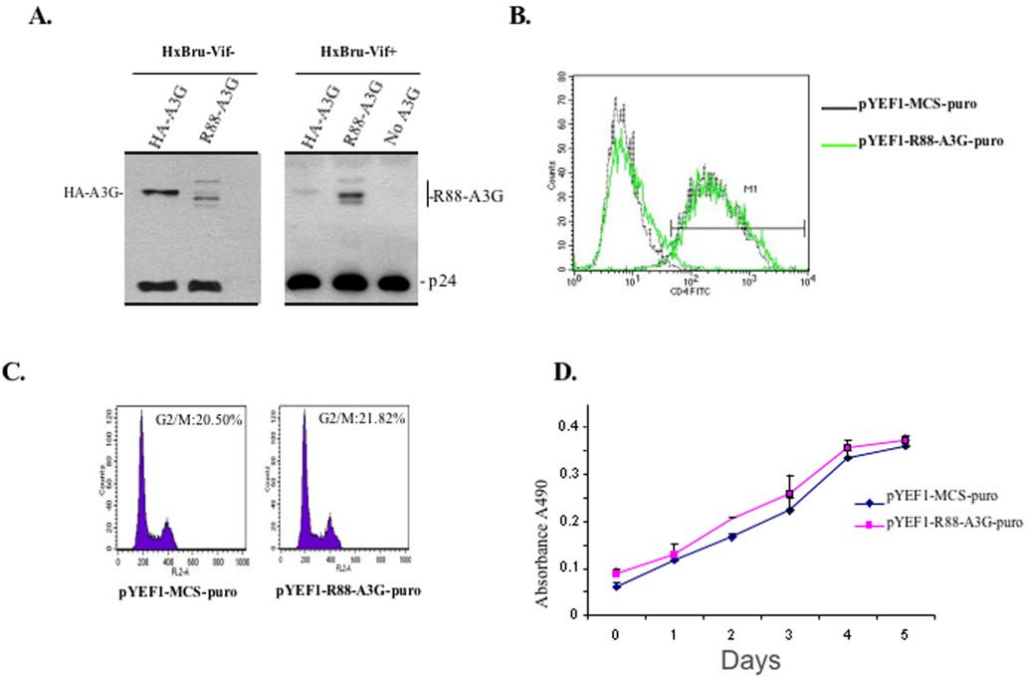
The R88-A3G is stably expressed in CD4+ C8166 cells and its presence does not affect cell growth and CD4 receptor expression. C8166 T cells (10×10^6^) were transduced with lentiviral vectors containing R88-A3G (pYEF1-R88-A3G-puro), HA-A3G (pYEF1-HA-A3G-puro) transgenes or empty vector (pYEF1-MCS-Puro), and puromycin-resistant cell population was selected by puromycin (0.5 µg/ml). Once the puromycin-resistant cell population was obtained, different analyses were performedfor their characterization. A). To detect R88-A3G and HA-A3G expression in C8166 T cells, transduced cells were infected with HxBru-Vif^−^ and HxBru-Vif^+^ virus. After 72 hours of infection, the produced viruses in the supernatant were concentrated by ultracentrifugation over 20% sucrose cushions. Then, virus pellets were lysed and resolved by 10% SDS-PAGE followed by Western blotting using anti-A3G and anti-p24 antibodies. B). The CD4 receptor expression levels observed on vector- and R88-A3G-transduced C8166 T cells were analyzed by using anti-CD4 staining and a flow cytometry assay. C). The cell cycle profile of vector- and R88-A3G-transduced C8166 T cells was analyzed by measurement of the cellular DNA content by staining with 30 µg/ml of propidium iodide (PI) and flow cytometry assay. D). To assess the growth of vector- and R88-A3G-transduced C8166 T cells, a WST assay was performed to determine cell viability at different time points. Each experiment was performed in triplicate and repeated at least three times. Results are shown as the mean ± SD of representative experiments.

Results from the above-mentioned experiments suggest that R88-A3G has effective antiviral activity when it is transiently over-expressed in 293T cells. We also studied whether stable low level expression of R88-A3G in CD4^+^ T cells exerts a similarly potent anti-HIV activity. First, we generated CD4^+^ C8166 T cell lines expressing either R88-A3G or HA-A3G fusion proteins by transducing C8166 T cells with a VSV-G pseudotyped lentiviral vector, which contained HA-A3G or R88-A3G transgene, as described in Materials and Methods. Briefly, lentiviral particles were produced by co-transfecting 293T cells with each lentiviral vector, HIV-1 Gag-Pol, Tat, and Rev plasmids and a VSV-G plasmid, as described previously [51]. To reduce the inhibitory effect of R88-A3G and HA-A3G on lentiviral vector transduction, we co-transfected the vector-producing with a pcDNA-hVif plasmid encoding a partially codon-optimized version of the native HIV-1 Vif gene [46]. CD4^+^ C8166 T cells were then transduced with the resultant lentiviral particles. Since the lentiviral vector contains a puromycin-resistance gene, the transduced cells were selected with puromycin (0.5 µg/ml) to obtain stable C8166 T cell lines expressing R88-A3G or HA-A3G. As expected, the transduction efficiency of lentiviruses containing R88-A3G was dramatically reduced (data not shown). However, through puromycin selection, we were able to obtain C8166 T cell lines stably expressing R88-A3G. The low levels of HA-A3G and R88-A3G protein present in the stably transduced cells were not detectable by anti-A3G Western blotting. However, we identified virion-incorporated HA-A3G or R88-A3G protein from highly concentrated HxBruVif^−^ and HxBruVif^+^ viruses produced from these two cell lines by immunoblotting with an anti-A3G antibody (Fig. 5A). Interestingly, results revealed that while abundant amounts of HA-A3G were present in Vif^−^ virus, there was a little HA-A3G detected in Vif^+^ viruses. In contrast, significant amounts of R88-A3G were detected in both Vif^−^ and Vif^+^ viral particles. These results suggest that the presence of Vif could not block R88-A3G's incorporation into HIV-1 particles, which are produced from C8166 T cell line.

Prior to studying the resistance of an R88-A3G expressing cell line to HIV-1 infection, we characterized the possible impact of stable expression of R88-A3G on cell growth and expression of an HIV receptor, CD4. CD4 surface receptor expression on R88-3G- and vector-transduced C8166 T cell lines were measured by anti-CD4 staining and FACS analysis. This analysis showed no difference between the two transduced C8166 T cell lines (Fig. 5B), indicating that low level R88-A3G expression had no detectable effect on CD4 surface receptor expression.

HIV-1 Vpr has been shown to induce cell cycle arrest at the G2/M phase [52]-[55]. Since R14-88 was derived from Vpr, we assessed whether R88-A3G affected cell division. We analyzed the cell cycle profile and growth kinetics of R88-A3G- and vector-transduced C8166 T cell lines by performing cell cycle analysis [56] and a cell proliferation (WST-1) assay, as described in Materials and Methods. No differences in the cell cycle profile or cell growth analysis were observed between R88-3G- and vector-transduced C8166 T cell lines (Fig. 5C and D).

### CD4^+^ C8166 T cells expressing R88-A3G are resistant to Vif^+^ HIV-1 infection

**Figure 6:**
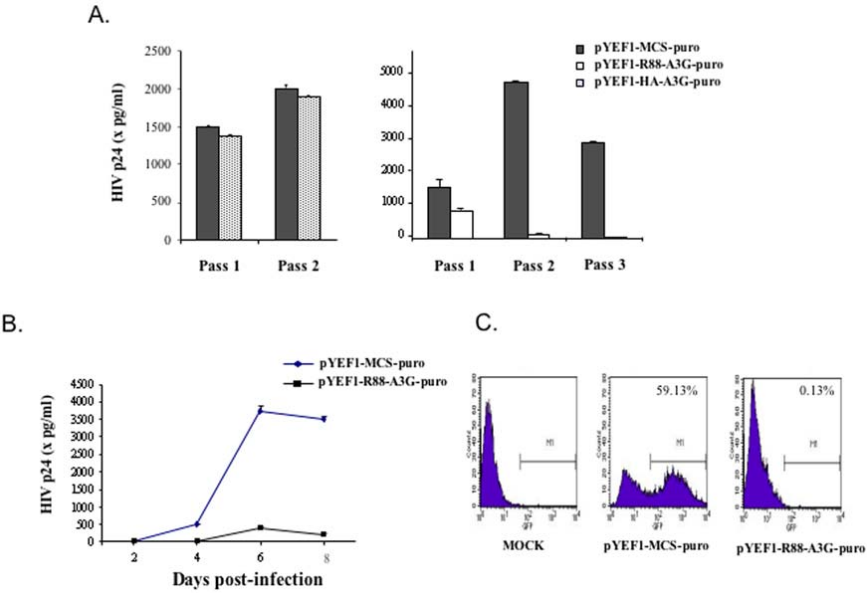
The resistance of an R88-A3G-transduced CD4+ C8166 T cell line against HIV-1 infection. A). Equal amounts of pNL4.3-GFP virus were used to infect vector-, HA-A3G-, or R88-A3G-transduced C8166 cell lines. After 48 hours of infection, virus-containing supernatants (passage 1) were collected, and the same volume of infectious supernatant was used to infect fresh and transduced C8166 cells (passage 2). The same procedure was performed again until virus-containing supernatants at assage 3 were obtained. Then, levels of HIV-1 Gag-p24 antigen in supernatants from passages 1, 2, and 3 were measured by HIV p24 ELISA assay. B). To test the effect of R88-A3G on virus infectivity, equal amounts (adjusted by Gag-p24 level) of virus from passage 1 were used to infect fresh R88-A3G-transduced C8166 cells. At different time points, the supernatants were collected and the HIV Gag-p24 level was measured to monitor virus replication. Also, at day 6 post-infection, the percentage of HIV-infected (GFP-positive) cells was evaluated by the flow cytometry assay (C).

We then assessed the resistance of R88-A3G-transduced C8166 T cells to HIV-1 infection. Vector-, HA-A3G- or R88-A3G-transduced cells (1×10^6^ cells) were infected with equal amounts of Vif^+^ HIV-1 virus (pNL4.3-GFP). At 72 hours post-infection, virus-containing supernatants (passage 1) were collected. Similar volumes (1 ml) of infectious supernatant were used to infect fresh and corresponding C8166 cell lines (passage 2). At 3 days post-infection, the same volumes (1 ml) of infectious supernatant were collected and used to infect corresponding C8166 cell lines (passage 3). The HIV-1 Gag-p24 levels in supernatants collected from corresponding cultures for each passage were measured by an HIV-1 p24 ELISA. The results of this assay indicate that initial infection of R88-A3G-transduced cells with Vif^+^ HIV-1 (passage 1) resulted in viral production that was approximately 50% of that observed for vector-transduced cells (Fig. 6A, right panel), while the viral replication in HA-A3G-transduced cells was similar to that observed in vector-transduced cells (Fig. 6A, left panel). This data suggests that R88-A3G-transduced C8166 T cells may exert certain level of resistance to incoming virions. Strikingly, viruses collected from R88-A3G-transduced C8166 T cells were unable to mediate subsequent infection in passages 2 and 3 (Fig. 6A), suggesting that virus transmission in R88-A3G-transduced cells was efficiently blocked (Fig. 6A, passages 2 and 3).

To rule out the possibility that the reduction or loss of virus transmission in the following passages may be a result of the reduced virus production, we evaluated viral replication kinetics in R88-A3G- or vector-transduced cells by infecting cells with equal amounts of the viruses, which were normalized by Gag-p24 levels produced by the corresponding cell lines (passage 1). At every 2-day interval, Gag-p24 ELISA and the FACS analysis were performed to assess virus replication. The results of these assays revealed that progeny viruses produced from R88-A3G-transduced cells lost their replication potential over an 8-day period (Fig. 6B and C). Together, this data indicates that R88-A3G-transduced C8166 cells are resistant to HIV-1 infection and that R88-A3G may prevent virus dissemination.

### Vif^+^ virus produced from R88-A3G-transduced CD4^+^ C8166 T cells loses infectivity in human primary mononuclear cells (PBMCs)

**Figure 7:**
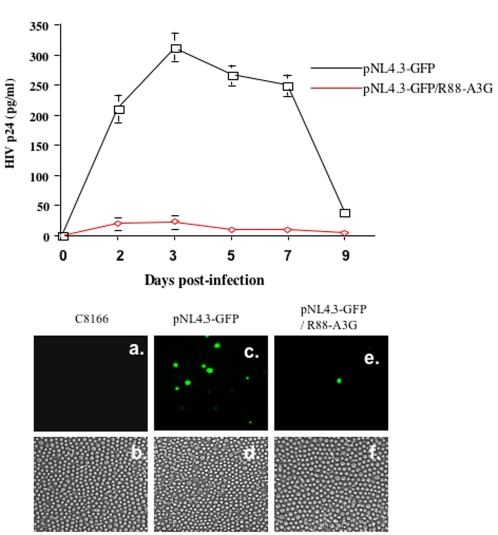
HIV-1 virus generated from CD4+ T cells stably expressing R88-A3G lost infectivity in human primary blood mononuclear cells (hPBMCs). Equal amounts of pNL4.3-GFP viruses produced from vector- or R88-A3G-transduced C8166 cells (in figure 6, passage 1) were used to infect 1×10^6^ PHA-stimulated human PBMCs for 8 hours. After infection, PBMCs were washed and at different time intervals, virion-associated Gag-p24 antigen levels in the supernatant were measured by anti-p24 ELISA (A). Values were representative of data obtained from two independent experiments. At 5 days post-infection, the numbers of infected (GFP-positive) cells in mock-infected, or differently HIV-1 infected hPBMC cells, as indicated, were visualized under fluorescence microscopy (B).

We assessed whether R88-A3G blocks HIV-1 infection in human PBMCs. Equal amounts of pNL4.3-GFP viruses produced from vector- or R88-A3G-transduced C8166 cells (in Fig. 6, passage 1) were used to infect 1×10^6^ PHA-stimulated human PBMCs. At 8 hours post-infection, the PBMCs were washed and cultured in medium supplemented with 5% IL-2. At the indicated time intervals, supernatants were collected and virion-associated Gag-p24 antigen levels were measured by anti-p24 ELISA (Fig. 7A). As expected, viruses produced from vector-transduced T cells productively infected human PBMCs. However, viruses generated from R88-A3G-transduced C8166 cells were not able to initiate an efficient infection, as indicated by the very low levels of p24 antigen detected in supernatants harvested up to 9 days post-infection (Fig. 7A). Similarly, fluorescence microscopy analysis indicated relatively large numbers of infected (GFP-positive) cells among the PBMCs infected with virus produced from vector-transduced T cells (Fig. 7B, c and d). In contrast, there were very few infected (GFP-positive) cells detected in the cell pools infected with viruses containing R88-A3G. In four observed fields, only one GFP-positive cell was found, which is shown in figure 7B (e and f). Together, these results clearly indicate that the presence of R88-A3G in Vif^+^ virions effectively blocks virus infection of human PBMCs.

## Discussion

The cellular cytidine deaminase A3G is a powerful innate antiviral factor that restricts HIV-1 infection in resting peripheral blood CD4^+^ T-lymphocytes [7]. However, during HIV-1 replication in activated T cells and macrophages, its action is counteracted by HIV-1 Vif protein [1]. Vif interacts with A3G and subsequently induces its degradation and prevent its incorporation into viral particles [6], [17]–[20]. Thus, determination of a mechanism whereby A3G may escape from Vif's blockage, which would rescue its potent antiviral activity, is of considerable interest, as it would provide a novel therapeutic strategy for treating HIV-1 infection. Here, we developed a strategy to target A3G into Vif^+^ HIV-1 particles by altering A3G's virion packaging pathway. A virus-targeting peptide (R14-88) derived from HIV-1 Vpr was fused to A3G. This peptide efficiently targets heterologous enzymatic proteins into HIV-1 particles in a p6-dependent manner [41]. We speculated that the R14-88 peptide may efficiently deliver A3G into HIV-1 particles, even in the presence of Vif. We placed an HIV-1 protease cleavage site between R14-88 and A3G to ensure that the native form of A3G is released by viral protease-mediated cleavage once the fusion protein is in the virus. Our results confirmed that R88-A3G was efficiently incorporated into Vif^+^ viruses and the majority of the fusion protein was converted to the native A3G form (Fig. 3B). More significantly, we demonstrated that R88-A3G expression efficiently inhibits HIV-1 infection in CD4^+^ cells and human PBMCs, regardless of the presence of Vif. These results provide clear evidence that the R88-A3G fusion protein is capable of overcoming Vif's blockage and significantly inhibiting HIV-1 replication.

It is known that Vif reduces the intracellular level of A3G in virus-producing cells by inducing rapid A3G degradation [17]–[19] and inhibiting its synthesis [17]. Further studies revealed that Vif recruits cullin 5-elongin B/C E3 ubiquitin ligase which polyubiquitinates A3G and results in its proteasomal degradation [20], [21], [57]. These findings have led to the prevailing opinion that Vif inhibits A3G encapsidation by inducing its rapid degradation in virus-producing cells. In addition, several other studies suggest that Vif may act as an effective barrier to sequester A3G and prevent its incorporation into virus, even though a low level of A3G and/or degradation-resistant mutants of A3G are detected in Vif^+^ HIV producing cells [6], [22], [24]–[26]. Accordingly, we wonder whether the higher level of R88-A3G in the virus could be due to the lower sensitivity of R88-A3G to Vif-induced degradation. Based on the results shown in figure 3A, it is clear that R88-A3G and HA-A3G are equally susceptible to Vif-mediated degradation. Consistent with previous observations, these data suggest that the intracellular degradation of A3G may not be the sole role played by Vif in the production of infectious viruses from A3G-expressing cells. An interaction between Vif and A3G may block virion-incorporation of A3G, which may be necessary for complete neutralization of A3G. In the case of R88-A3G, the incorporation pathway of the fusion protein is different from that of A3G. This altered incorporation pathway can bypass Vif's blockage, and consequently restore A3G's antiviral activity. However, at this point, we do not know whether the efficient virion-incorporation ability of R88-A3G is the only factor that accounts for its potent anti-HIV activity, because a low level of HA-A3G was detected in Vif^+^ HIV virus lysates (Fig. 3B), while they were not functional (Fig. 2A and B). We speculate that, in addition to its ability to target A3G into virus particles, R14-88 may further optimize A3G's activity, possibly by altering its intraviral localization or its association with viral components. Indeed, a recent study by Aguinar *et al* suggested that Vpr targeted A3A (APOBEC3A), another member of the human APOBEC3 protein family, into the viral core and possibly into the ribonucleoprotein complex (RNP), but that A3A alone was only loosely associated with viral particles [58]. It is possible that, in the presence of Vif, R88-A3G is targeted to the core and associated with the RNP, which is critical for the inhibitory effect of R88-A3G. In contrast, Vif may disrupt the intraviral location of native A3G, including HA-A3G, even though a small amount of HA-A3G may be packaged in the virus. We posit that these questions merit further study.

The inhibition of R88-A3G on Vif^+^ virus infectivity was examined by measuring its effect on viral reverse transcription synthesis. Viruses produced by R88-A3G-expressing cells were defective at reverse transcription (Fig. 4B), which closely correlated with the loss of viral infectivity (Fig. 2). These observations indicate that one of main functions of R88-A3G is to directly affect HIV-1 reverse transcription, which is similar to the activity of native A3G during Vif^−^ virus infection. Indeed, several previous studies have shown that Vif^−^ viruses produced from A3G expressing cells produced a severely reduced level of viral DNA in newly infected cells [59]–[62]. In addition, a report by Bishop *et al*. revealed that the antiviral phenotype of APOBEC proteins correlates with their ability to prevent accumulation of reverse transcripts, but not with the induction of hypermutations [47]. Although R88-A3G significantly inhibits the reverse transcription step, it is worth noting that A3G's deaminase activity may contribute to the potent antiviral effect of the R88-A3G fusion protein. Further study is underway to assess the contribution of A3G deaminase activity to R88-A3G's activity, and to evaluate its contribution to the mutational burden carried by viral DNAs.

Our initial experiments revealed that co-expression of R88-A3G and HIV-1 in 293T cells significantly inhibited progeny virus infectivity regardless of the presence of Vif protein. Importantly, these experiments raise the concern that the effect of R88-A3G is, at least in part, due to its over-expression during viral production. Therefore, it is necessary to assess whether a lower level of R88-A3G expression in CD4^+^ T cells renders cells resist to viral infection. To that end, we introduced R88-A3G into CD4^+^ C8166 T cells by use of a lentiviral vector. These transduced CD4^+^ T cell lines expressed very low levels of the R88-A3G fusion protein, such that we could not detect R88-A3G protein expression in these cells. Instead, we detected R88-A3G in concentrated viruses released from these cells (Fig. 5A). Our infection experiments consistently demonstrate that a low level of R88-A3G in C8166 T cells sufficiently inhibits Vif^+^ HIV-1 infection and transmission (Fig. 6). It should also be noted that stable R88-A3G expression did not affect C8166 T cell growth, cell division or CD4 surface expression (Fig. 5B, C and D). Interestingly, our experiments revealed that virus production was partially reduced in R88-A3G-transduced C8166 T cells initially infected with Vif^+^ virus, while HA-A3G-transduced cells were fully susceptible to viral infection (Fig. 6A). This is surprising since A3G is generally observed with a high molecular mass (HMM) in activated T cells, which is not able to affect virus replication during the first round of infection [7], [63]. At this point, we do not know whether the R88-A3G fusion protein has undefined features that may affect incoming virus replication. A previous study has shown that adding recombinant Vpr could *in trans* complemented HIV-1 preintegration complex for its nuclear translocation in an *in vitro* nuclear import assay [64]. Therefore, whether the R14-88 peptide component of R88-A3G directs the fusion protein to incoming virus reverse transcription complexes and consequently affects early stages of viral replication is worthy of further investigation. Another interesting observation is that the progeny virus produced from R88-A3G-transduced C8166 T cells lost their ability to mediate subsequent infection (Fig. 6A), indicating that Vif^+^ virus is not transmissible in the presence of R88-A3G.

Overall, this study demonstrates a novel strategy to modify a host innate anti-HIV protein and rescue its potent anti-HIV potential, even in the presence of Vif. Further characterization and optimization of this system may lead to development of an effective therapeutic approach against this deadly viral infection.

## Materials and Methods

### Plasmid construction

The cDNA of human APOBEC3G (A3G) was kindly provided by Dr. S.K. Petersen-Mahrt [65], [66]. To generate the CMVin-R14-88-pcs-APOBEC3G (CMVin-R88-A3G) fusion protein expressor, a cDNA was first amplified by polymerase chain reaction (PCR), which encodes for an initiation codon (ATG) followed by amino-acids from 14 to 88 of HIV-1 Vpr and a HIV-1 protease cleavage site. The primers used for the PCR are as follows: 5′ oligo: 5′-GCGGGATCCAGAGGATAGATGCCACACAATGAA-3′, and the 3′oligo: 5′-GCTCTAGATCCACCTACGATCGGATAATTCTGGCTTACTCTCCTCTG-3′. At the 5′ and the 3′ ends of cDNA, a *BamHI* and a *Xba*I site were created, respectively, and used to clone the cDNA into an SVCMVin vector [67]. The constructed plasmid was named CMVin-R14-88-pcs. Then, a full-length wild-type A3G cDNA was amplified by PCR using the 5′-A3G-*Xba*I primer (5′-TCATCTAGAAAGCCTCACTTCAG-3′) and 3′-*Bgl*II primer (5′-TAAGAGCTCATGCCTGCAGCC-3′). The amplified A3G cDNA was cloned into CMVin-R14-88-pcs, in frame to the 3′ end of R14-88-pcs, and the plasmid was named CMVin-R88-A3G. Also, a hemagglutinin (HA)-tagged A3G expression plasmid (CMVin-HA-A3G) was constructed by first fusing the A3G cDNA to 3′ end of the cDNA encoding for the HA sequence (MASYPYDVPDYASL) by PCR and the amplified cDNA was cloned into the SVCMVin vector. The HIV-1 Vif expressor (pcDNA-hVif) [46] was obtained from the NIH AIDS Research and Reference Reagent Program. The HIV-1 proviral clones HxBru-Vif^+^ [32] and pNL4.3-Nef^+^/GFP/Vif^+^ (pNL4.3-GFP) [68] were kindly provided by Dr. E. Cohen. To construct a HxBru-Vif^−^ provirus, a two-step PCR technique, which was described previously [32], was used to amplify a cDNA encompassing a region between *Apa*I and *Sal*I sites of provirus, in which amino-acids at positions 21 and 22 of Vif were changed to a stop codon. Then, the amplified PCR fragment was digested with *Apa*I and *Sal*I restriction enzymes and was used to replace the corresponding region in HxBru-Vif^+^, and the resultant provirus was called HxBru-Vif^−^. The sense mutagenic primer is 5′-GAGGATTAGAACATGATGACGTGTAGTAAAACACC-3′.

To generate a lentiviral vector for T cell transduction, a pEF1-pcs-puro vector was first constructed based on a HIV-based vector pHxEGFPWP [51]. Briefly, a region between *SnaB*I (nt 8328) and *Kpn*I (nt 10110) in pHxEGFPWP was replaced by PCR-amplified cDNAs containing two EF1 promoters, the first one followed by a *BamH*I/*Nhe*I/*Cla*I cloning site and the second promoter linked with a puromycin gene with a Woodchuck hepatitis virus posttranscriptional regulatory element (WPRE). These cDNAs were amplified from the lentiviral vector pCDH1-MCS1-EF1-puro, which was purchased from System Biosciences (SBI). After the pYEF1-pcs-puro vector was generated, cDNAs encoding for R88-A3G or HA-A3G were cloned into this vector through *BamH*I/*Cla*I or *Nhe*1/*Cla*I sites, and the resultant plasmids were named pYEF1-R88-A3G-puro and pYEF1-HA-A3G-puro.

#### Cell lines and transfection

Human embryonic kidney 293T cells and HeLa-CD4/CCR5 β-Gal cells were maintained in Dulbecco's Modified Eagles Medium (DMEM) supplemented with 10% fetal calf serum (FCS) and 1% penicillin and streptomycin. The CD4^+^ C8166 T cell lines were maintained in RPMI-1640 medium containing 10% FCS and 1% penicillin and streptomycin PBMCs were isolated from the blood of healthy adult volunteers by sedimentation in Ficoll-Hypaque (Sigma-Aldrich Canada, Inc., Oakville, Ontario). Isolated PBMCs were stimulated with 0.1% phytohemaglutinin (PHA) and maintained in RPMI 1640 supplemented with 5% IL-2. DNA transfection of 293T cells was performed with standard calcium phosphate DNA precipitation method.

#### Antibodies and chemicals

Antibodies used in immunoprecipitation or Western blotting are as follows. The purified rabbit anti-hA3G (Cat# 10201), anti-RT (Cat# 6195) and anti-Vif (Cat#2221) polyclonal antisera were obtained through the NIH AIDS Research and Reference Reagent Program. The anti-HA monoclonal antibody was purchased from Sigma. The rabbit anti-Vpr antibody and HIV-1 positive human serum 162 were described previously [32]. Mouse anti-HIVp24 and anti-CD4 monoclonal antibodies used in this study were described previously [50], [69].

#### Virus production and infection

To test the effect of R88-A3G and HA-A3G on HIV-1 infection, 293T cells were co-transfected with corresponding HIV-1 proviral DNA and R88-A3G or HA-A3G plasmids. Supernatants were collected at 48 hours post-transfection and subjected to ultra-centrifugation (32,000 rpm for 1 hour at 4°C) to pellet the virus. Quantification of virus stocks was determined by Gag-p24 measurements using an HIV-1 p24 ELISA Kit (purchased from the AIDS Vaccine Program of the Frederick Cancer Research and Development Center) or by an RT activity assay [70]. For immunoblot analysis, viruses were pelleted by ultracentrifugation through a 20% sucrose cushion. Then, the lysed virus samples were analyzed by SDS-PAGE followed by Western blotting.

To evaluate virus infection in HeLa-CD4/CCR5-β-Gal cells, equal amounts (adjusted by Gag-p24 levels) of virus were used to infect cells in 24-well plates. At 48 hours post-infection, the number of β-Gal positive cells was detected by the MAGI assay, as described previously [45], and counted with an Elispot Reader (SID Autoimmune Diagnostika GmbH).

To infect CD4+ C8166 T cells and human PBMCs, equal amounts of viruses were incubated with susceptible cells at 37°C for 4 hours. Then, the cells were washed and incubated with fresh medium. At different time points, viral production levels were monitored by measurement of HIV-1 Gag-p24 antigen in each infected culture supernatant by HIV-1 Gag-p24 ELISA. To evaluate the infection mediated by pNL4.3-GFP HIV-1 virus, infected cells were fixed with PBS-4% paraformaldehyde and detected by fluorescence-activated cell sorter (FACS; Becton Dickenson FACS Calibur) analysis or observed under fluorescence microscopy.

#### Establishment of C8166-R88-A3G or C8166-HA-A3G cell lines

Production of VSV-G pseudotyped lentiviral vectors: Pseudotyped lentiviral vector stocks were produced by co-transfection of 293T cells with pYEF1-R88-A3G-puro or pYEF1-HA-A3G-puro vector, the HIV packaging plasmid pCMVΔR8.2 [51] and VSV-G expression plasmids. As a control, the pYEF1-mcs-puro vector was used to produce a lentiviral vector. To reduce the incorporation of R88-A3G into the vector particles, the pcDNA-hVif plasmid was co-transfected with pYEF1-R88-A3Gpuro (molar ratio 3:1). After 48 h of transfection, the vector particles (VPs) from different supernatants were concentrated by ultracentrifugation (at 32,000rpm) for 1.5 hour at 4°C.

Transduction and puromycin selection: VPs (equivalent to 100 to 300 ng of Gag-p24) were incubated with C8166 T cells for overnight. After washing, transduced and non-transduced cells were incubated with fresh RPMI containing puromycin (0.5 µg/ml). The transduced C8166 T cells were under selection of puromycin for at least 10 days. As a control, non-transduced cells were likewise submitted to selection and did not survive beyond day 4 under the same concentration of puromycin. The cell cycle profile of transduced cells was analyzed as previously described [70]. The CD4 receptor expression of different transduced C8166 T cells was evaluated by anti-CD4 staining and analyzed by a fluorescence-activated cell sorter (FACS, Becton Dickenson FACS Calibur). Briefly, cells were incubated with mouse anti-CD4 (OKT4) at 4°C for 2 hours; the cells were then washed and stained with anti-mouse IgG-FITC in the dark for 30 min at 4°C. Cells were then fixed with 4% paraformaldehyde and analyzed by FACS analysis.

Cell proliferation assay: The number of surviving cells was measured by the 4-[3-(4-iodophenyl)-2-(4-nitrophenyl)-2H-5-tetrazolio]-1,3-benzene disulfonate (WST-1) assay (Roche). Briefly, pYEF1-MCS-puro and pYEF1-R88-A3G-puro transduced cells were cultured at a density of 15×10^3^ cells/well in 96-well plates and incubated at 37°C. At different time points, WST (10 µl/well) was added in the culture and the cells were incubated for 4 hours at 37°C. After shaking thoroughly for 1 min, the absorbance was measured at 490 nm using a microplate (ELISA) reader.

#### Radiolabeling, immunoprecipitation and Western blot analyses

To perform pulse-chase radiolabeling experiments, 293T cells were transfected with HA-A3G or R88-A3G expressor alone or co-transfected with pcDNA-hVif expressor. After 40 hours, cells were incubated for 30 min with starve medium (DMEM without methionine, plus 10% dialyzed FBS). Then the cells were pulse-labeled for 30 min with 200 µCi of [_35_S]-methionine (Perkin Elmer). At the end of labeling, the radiolabeled medium was removed, and cells were washed with medium containing an excess of un-labeled methionine and incubated in complete DMEM for various times at 37°C. Labeled cells were then pelleted, lysed and immunoprecipitated using human anti-HA or anti-Vpr antibody. Immunoprecipitates were then resolved by 10% SDS-PAGE followed by autoradiography.

To analyze protein expression in cells and/or in viral particles, cell lysates or lysed viral samples were directly loaded onto 10% SDS-PAGE gel and different protein levels were analyzed by Western blot with corresponding antibodies. The HRP-conjugated donkey anti-rabbit IgG, sheep anti-mouse IgG and anti-human IgG (Amersham Biosciences) were used as secondary antibodies, and protein bands were then visualized by using an enhanced chemiluminescence kit (PerkinElmer Life Science, Boston, MA).

#### HIV-1 reverse-transcribed DNA detection by PCR

C8166 T cells were infected with equal amounts of HxBru-Vif^+^ viruses produced from 293T cotransfected with HA-A3G or R88-A3G. Prior to infection, viruses were treated with 340U/ml DNAse (Invitrogen, Inc.) for 1 hour in 37°C to remove residual plasmid DNA. After 2 hours of infection, cells were washed with PBS and cultured in RPMI medium. At 12 hours post-infection, equal numbers (1×10^6^ cells) of cells were collected, and DNA was extracted using a QIAmp DNA Blood Mini kit, following the manufacturer's instructions (QIAGEN, Valencia, Calif.). The DNA samples were processed for detecting total viral DNA synthesis by using LTR-Gag primers, as described previously [50]. To evaluate the DNA content of extracted chromosomal DNA preparations, detection of human β-globin gene was carried-out by PCR, as described previously [71]. All final PCR products were electrophoresed through 1.2% agarose gels.
